# Harnessing the potential of digital data for infectious disease surveillance in sub-Saharan Africa

**DOI:** 10.1093/eurpub/ckac131.569

**Published:** 2022-10-25

**Authors:** J Boenecke, J Brinkel, M Belau, M Himmel, J Ströbele

**Affiliations:** 1 Department of Health Sciences, Hamburg University of Applied Sciences, Hamburg, Germany; 2 Department Infectious Disease Epidemiology, Bernhard Nocht Institute for Tropical Medicine, Hamburg, Germany; 3 Institute of Medical Biometry and Epidemiology, University Medical Center Hamburg- Eppendorf, Hamburg, Germany; 4 Department for Microbiology and Biotechnology, Institute for Plant Sciences and Microbiology, University of Hamburg, Hamburg, Germany; 5 Department of Computer Science, Hamburg University of Applied Sciences, Hamburg, Germany

## Abstract

Despite efforts by the WHO to support local surveillance strategies in developing countries, there is a lack of robust public health surveillance frameworks. As a result, early infectious disease outbreak detection and response remain a significant challenge for local health systems in low-resource settings such as sub-Saharan African countries. In contrast, the growing digital infrastructure, especially in the mobile phone sector, and the global availability of extensive digital data offer promising solutions to enhance and strengthen epidemiological surveillance. Yet, there is little insight into concepts of utilisation and transfer into local public health practice. Using Tanzania as an example, a novel electronic surveillance and early outbreak alert framework is being developed that links signals on emerging diseases with relevant contextual Open Data for rapid outbreak risk assessment. The concept focuses on haemorrhagic fever diseases, specifically dengue virus disease, which is increasingly spreading in sub-Saharan Africa. A data stack framework forms the core of the system, which augments electronic information on the occurrence of acute haemorrhagic fever syndrome, e.g., collected via mobile phone-based surveillance tools, with openly available socio-ecological context data specific to dengue. Preliminary results on the data and information flow within the surveillance framework are presented and strategies for an automated indicator-based risk assessment for dengue outbreaks will be discussed, supplemented by an agent-based simulation framework to model possible short-term outbreak scenarios. In addition, adequate data inputs, identified through an appraisal of various data sources available for Tanzania, are outlined. The framework could serve as a blueprint for designing locally implementable early warning and decision support systems integrated with existing digital surveillance infrastructure.

**Key messages:**

• Digital health surveillance and Open Data offer great potential for early outbreak detection and supporting health decisions but require tailored solutions to benefit low-resource settings.

• Building on existing digital surveillance infrastructure, the framework may serve as a blueprint for designing an enhanced surveillance and decision support system for infectious disease outbreaks.

